# Initial experience of transurethral ultrasound ablation of the prostate in Asia

**DOI:** 10.1002/bco2.175

**Published:** 2022-06-23

**Authors:** Masayoshi Miura, Satoru Takahashi, Maiko Fukumoto, Hiroshi Higashiyama

**Affiliations:** ^1^ Department of Urology Sapporo Hokuyu Hospital Sapporo Japan; ^2^ Imaging Research Center Takatsuki General Hospital Takatsuki Japan; ^3^ Transplant Medical Support Office Sapporo Hokuyu Hospital Sapporo Japan

**Keywords:** image‐guided therapy, minimally invasive, MRI, prostate cancer, thermal ablation, transurethral MR‐guided ultrasound

1

Transurethral ultrasound ablation of the prostate (TULSA) is a novel modality for the treatment of localised prostate cancer.^1^, ^2^, ^3^, ^4^ TULSA uses linear high‐frequency ultrasound to heat the prostatic tissue, and the treatment margin is precisely controlled by closed‐loop control of ultrasound emission by measuring the temperature of prostatic tissue inside a magnetic resonance imaging (MRI) bore in a real‐time manner. The surface of the urethra and the rectal wall are cooled during the treatment.^1^, ^2^, ^3^, ^4^ The pivotal trial in Europe and North America showed significantly low rates of urinary incontinence and erectile dysfunction without compromising cancer control for low and intermediate‐risk cancer.^4^ We herein show our initial experience of TULSA for the first time in Asia.

Five men with localised prostate cancer who underwent TULSA and followed for over 1 year are included. There were 4 cases of high‐risk cancer and one case of intermediate‐risk cancer. Two men underwent whole‐gland ablation and three partial ablation. Patients' characteristics and representative MRI images are shown in Table S1 and Figure S1. A Foley catheter was placed after the procedure and was removed the next morning. Patients underwent clean intermittent catheterization (CIC) in case of voiding difficulty. Changes in prostate‐specific antigen (PSA) levels, MRI findings at 6 and 12 months after TULSA, prostatic volume measured by MRI, changes in symptoms and quality of life (QOL) using Expanded Prostate Cancer Index Composite^5^ and SF‐12 Health Survey^6^ were recorded.

All patients underwent uneventful TULSA and returned to normal activity immediately. Two patients with whole‐gland and one patient with subtotal ablation underwent self CIC for 7–17 days, and two with partial ablation did not require CIC. All patients were pad‐free after TULSA. Erectile and ejaculatory functions were preserved in the one patient who was sexually active before TULSA. No complications were observed. PSA remained stable for up to 1 year in four patients (Figure 1). These 4 patients showed negative MRI studies at 6 and 12 months. The mean prostate volume of 5 patients decreased by 47% from 24 mL pre‐TULSA to 12 mL at 1 year. One patient with the whole‐gland therapy showed PSA relapse at 10 months, and MRI showed tumour recurrence involving the left seminal vesicle with no evidence of metastasis. He did not undergo an MRI before referral and had already been under androgen deprivation therapy (ADT) for 6 months at referral. He was assumed to have had seminal vesicle invasion originally, which was outside the ablation boundary. He underwent a successful salvage radiation therapy of 70Gy and ADT, because TULSA does not preclude any salvage treatments.

**FIGURE 1:**
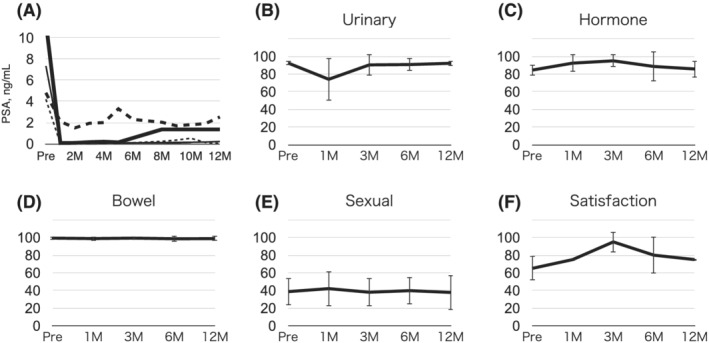
(A) Changes in prostate‐specific antigen (PSA) levels. The thin broken line represents the case with PSA relapse. The thick broken line represents the case of partial ablation. The thick solid line also represents the case of partial ablation who underwent androgen deprivation therapy before transurethral ultrasound ablation of the prostate (TULSA). (B–F) Expanded Prostate Cancer Index Composite average scores. Note that urinary score dropped at 1 M due to voiding difficulty but recovered thereafter. There was no significant change in hormone or bowel scores. Four patients were sexually inactive before TULSA, and average scores are low. Satisfaction score was kept high throughout the period. The bars denotes standard error.

SF‐12 survey showed no significant changes in component summary scores throughout the study period, and the average scores were higher than the Japanese average.

We herein showed our initial experience of TULSA in Japanese patients. Cancer control up to 1Y was satisfying in 4 patients. One of the important features of TULSA is that it has a very low rate of urinary incontinence, which has been the most significant unsolved problem after radical prostatectomy.^7^ Very accurate control of the treatment boundary brings secure ablation of the tissue to the boundary with no damages beyond, as all the patients in our series were continent after TULSA. One patient requested the preservation of erectile and ejaculatory functions, both of which were successfully preserved by unilateral partial ablation while excluding the ejaculatory ducts. Accurate margin control offers a great advantage for patients who seek for retaining their function. TULSA is an incision‐free therapy, which can be performed on a single day with a short‐term hospitalisation. No decline in QOL was observed, and patients' satisfaction score was considerably high. The prostate swells after heat ablation and the prostatic urethra can be obstructed especially after a whole‐gland treatment. In other TULSA trials, a suprapubic catheter was placed during treatment.^1^, ^4^ However, we applied self‐CIC temporarily for the patients who developed voiding difficulty because we consider CIC is less invasive for the post‐treatment management of urination.

Currently, non‐metastatic cancer with stage ≤T2c is the inclusion criteria for TULSA in our centre. Low‐ to intermediate‐risk, organ‐confined prostate cancer best suited to TULSA. Focal ablation has created opportunities for functional preservation in prostate cancer treatment, as shown here with the two focal ablations. However, TULSA monotherapy may be insufficient for T3 cancer because the treatment range of TULSA is 3 cm from the urethra, and the seminal vesicles are usually beyond the range. Additionally, the venous plexus surrounding the prostate may produce a cooling effect resulting in insufficient heating of extracapsular invasion. Despite these limitations, TULSA may have a role in multimodal therapy for high risk cancer and awaits future research.

Our early experience shows that TULSA is safely applicable for treating localised prostate cancer, with a minimal impact on patients' QOL.

## CONFLICT OF INTEREST

Author M.M. is engaged in medical consultant for Profound Medical Inc. Profound Medical Inc. has no role in the design, practice or analysis of this study.

## Supporting information


**Figure S1.** Representative MRI images showing tumour foci.
MRI images showing tumour foci prior to treatment are shown for Patients 1, 2, 3 and 5. For Patient 4, tumour foci were not identifiable in the MRI images prior to treatment since he was already on androgen deprivation treatment and MRI images at the time of tumour relapse are shown. Arrowheads indicate tumour foci.

**Table S1.** Baseline characteristics and treatment parameters

## References

[1] Chin JL , Billia M , Relle J , Roethke MC , Popeneciu IV , Kuru TH , et al. Magnetic resonance imaging‐guided transurethral ultrasound ablation of prostate tissue in patients with localized prostate Cancer: a prospective phase 1 clinical trial. Eur Urol. 2016;70(3):447–55. 10.1016/j.eururo.2015.12.029 26777228

[2] Chopra R , Tang K , Burtnyk M , Boyes A , Sugar L , Appu S , et al. Analysis of the spatial and temporal accuracy of heating in the prostate gland using transurethral ultrasound therapy and active MR temperature feedback. Phys Med Biol. 2009;54:2615–33. 19351975 10.1088/0031-9155/54/9/002

[3] Chopra R , Colquhoun A , Burtnyk M , Ndjin WA , Kobelevskiy I , Boyes A , et al. MR imaging‐controlled transurethral ultrasound therapy for conformal treatment of prostate tissue: initial feasibility in humans. Radiology. 2012;265:303–13. 10.1148/radiol.12112263 22929332

[4] Klotz L , Pavlovich CP , Chin J , Hatiboglu G , Koch M , Penson D , et al. Magnetic resonance imaging‐guided transurethral ultrasound ablation of prostate Cancer. J Urol. 2021;205(3):769–79. 10.1097/JU.0000000000001362 33021440

[5] Takegami M , Suzukamo Y , Sanda MG , Kamoto T , Namiki S , Arai Y , et al. Nihon Hinyokika Gakkai Zasshi. 2005;96:657–69. 10.5980/jpnjurol1989.96.657 16363651

[6] Ware J Jr , Kosinski M , Keller SD . A 12‐item short‐form health survey: construction of scales and preliminary tests of reliability and validity. Med Care. 1996;34:220–33. 8628042 10.1097/00005650-199603000-00003

[7] Potosky AL , Davis WW , Hoffman RM , Stanford JL , Stephenson RA , Penson DF , et al. Five‐year outcomes after prostatectomy or radiotherapy for prostate cancer: the prostate cancer outcomes study. J Natl Cancer Inst. 2004;96:1358–67. 15367568 10.1093/jnci/djh259

