# Supplementary figures and images for: Initial experience of transurethral ultrasound ablation of the prostate in Asia
Source: BJUI Compass. 2022 Jun 23;3(6):405–7. doi: 10.1002/bco2.175 (PMC9579879; doi:10.1002/bco2.175)

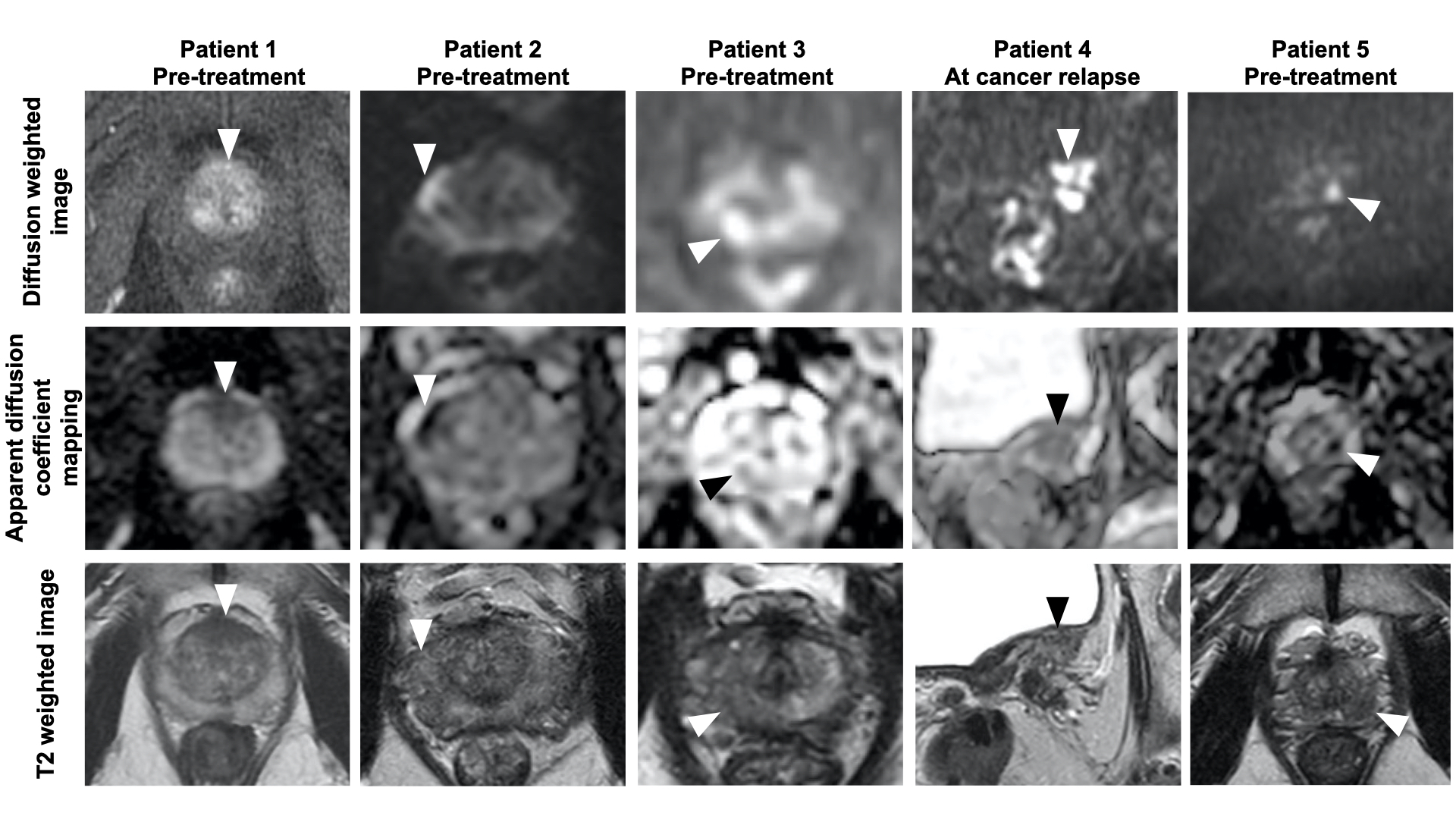

Supplement: Supplementary file 1 — Figure S1. Representative MRI images showing tumour foci. MRI images showing tumour foci prior to treatment are shown for Patients 1, 2, 3 and 5. For Patient 4, tumour foci were not identifiable in the MRI images prior to treatment since he was already on androgen deprivation treatment and MRI images at the time of tumour relapse are shown. Arrowheads indicate tumour foci. [file BCO2-3-405-s002.jpeg]
