# Supplementary material for: Initial experience of transurethral ultrasound ablation of the prostate in Asia
Source: BJUI Compass. 2022 Jun 23;3(6):405–7. doi: 10.1002/bco2.175 (PMC9579879; doi:10.1002/bco2.175)
Supplement: Supplementary file 2 — Table S1. Baseline characteristics and treatment parameters [file BCO2-3-405-s001.docx]

Supplement Table 1. Baseline characteristics and treatment parameters

| **Case No** | age | T stage | PI-RADS score in pre-treatment MRI | Grade Group | No of positive cores / Total no of sampled cores | Pre-TULSA PSA (ng/mL) | NCCN risk category | Length of pre-TULSA androgen deprivation therapy | Ablation type |
| --- | --- | --- | --- | --- | --- | --- | --- | --- | --- |
| **1** | 74 | T2c | 5 | 2 | 2/16 | 11.4 | high | 6M | focal |
| **2** | 68 | T2a | 5 | 5 | 1/14 | 11.5 | high | 2M | whole |
| **3** | 50 | T2a | 4 | 3 | 1/12 | 5.1 | intermediate | - | focal |
| **4** | 82 | T2c | - | 4 | 6/12 | 4.2 | high | 6M | whole |
| **5** | 71 | T2c | 5 | 3 | 8/20 | 6.9 | high | - | subtotal |

PI-RADS, prostate imaging reporting and data system

TULSA, transurethral ultrasound ablation of the prostate

MRI, magnetic resonance imaging
